# Novel Mutations in *FKBP10* and *PLOD2* Cause Rare Bruck Syndrome in Chinese Patients

**DOI:** 10.1371/journal.pone.0107594

**Published:** 2014-09-19

**Authors:** Peiran Zhou, Yi Liu, Fang Lv, Min Nie, Yan Jiang, Ou Wang, Weibo Xia, Xiaoping Xing, Mei Li

**Affiliations:** Department of Endocrinology, Key Laboratory of Endocrinology of Ministry of Health, Peking Union Medical College Hospital, Peking Union Medical College, Chinese Academy of Medical Sciences, Beijing, China; Innsbruck Medical University, Austria

## Abstract

Bruck syndrome (BS) is an extremely rare form of osteogenesis imperfecta characterized by congenital joint contracture, multiple fractures and short stature. We described the phenotypes of BS in two Chinese patients for the first time. The novel compound heterozygous mutations c.764_772dupACGTCCTCC (p.255_257dupHisValLeu) in exon 5 and c.1405G>T (p.Gly469X) in exon 9 of *FKBP10* were identified in one proband. The novel compound heterozygous mutations c.1624delT (p.Tyr542Thrfs*18) in exon 14 and c.1880T>C (p.Val627Ala) in exon 17 of *PLOD2* were identified in another probrand. Intravenous zoledronate was a potent agent for these patients, confirmed the efficacy of bisphosphonates on this disease. In conclusion, the novel causative mutations identified in the patients expand the genotypic spectrum of BS.

## Introduction

Osteogenesis imperfecta (OI) is a remarkably heterogeneous monogenic disease characterized by bone fragility and low bone mass, more than 90% of which are caused by dominant mutations in encoding genes of type I procollagen chains proα1 (*COL1A1*, MIM 120150) or proα2 (*COL1A2*, MIM 120160) [1], [2]. Bruck syndrome (BS, MIM 259450 and 609220) is an extremely rare autosomal recessive form of OI, which is defined by congenital large joint contracture, bone fragility, multiple bone fractures starting in infancy or early childhood [2]–[4]. According to the latest genetic research, mutations of genes encoding new proteins involved in post translational modifications and folding of type I collagen have been identified to give rise to autosomal recessive forms of OI, including *CRTAP, LEPRE1, PPIB, SERPINF1, BMP1, WNT1, TMEM38B*. *FKBP10* (MIM 607063) and *PLOD2* (MIM 601865), encoding FKBP65 and lysyl hydroxylase 2 (LH2), are also reported as targeted genes of the recessive forms of OI [1], [5].

FKBP65 is a rough endoplasmic reticulum resident protein which belongs to the family of prolyl *cis–trans* isomerases. FKBP65 acts as an important collagen chaperone-like protein participating in folding of type I procollagen [6]. LH2 is the key enzyme responsible for hydroxylation of lysine residues outside the major triple helix of type I collagen, crucial to formation of mature intermolecular cross-links in bone and cartilage [5], [7]. BS is caused by mutations of *FKBP10* or *PLOD2* [6]–[11]. According to different causative genes, BS is delineated into phenotypically indistinguishable type 1 (BS1) and type 2 (BS2), which are caused by mutations of *FKBP10* and *PLOD2*, respectively [7], [9], [11]. Genetic and phenotypic heterogeneity of BS has not been completely recognized. Up to now, only 48 individuals with *FKBP10* mutations and 10 individuals with *PLOD2* mutations have been reported all over the world, however, no information of BS is reported in Chinese.

Therefore, we identified the mutations of causative genes, described the clinical phenotype and evaluated the effects of bisphosphonates (BPs) for the first time in two new patients with BS.

## Materials and Methods

### Affected Family and Control

Two Chinese patients from different non-consanguineous families were diagnosed as BS in the department of endocrinology, Peking Union Medical College Hospital (PUMCH) from 2012 to 2013.

### Family 1

**Figure 1:**
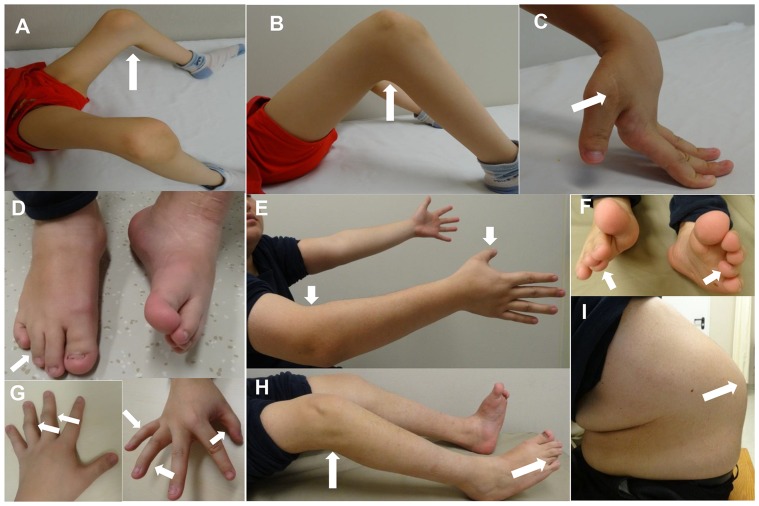
Clinical phenotypes in probands with Bruck syndrome. For proband 1, (A)–(B) severe flexion deformity of knees (white arrows); (C) small joint laxity of hand and scar of orthopedic surgery for correcting thumb-in-palm deformity (white arrow). For proband 2, (D) left pes equinovarus and invisible right fourth toe because of camptodactyly (white arrow); (E) congenital joint contracture of right elbow and camptodactyly of right thumb (white arrows); (F) camptodactyly of fourth toes (white arrows); (G) mild camptodactyly of left 3–4th fingers and right thumb, 4–5 fingers (white arrows); (H) limited movement of knees and invisible right fourth toe from this view (white arrows); (I) severe ithyokyphosis.

**Figure 2:**
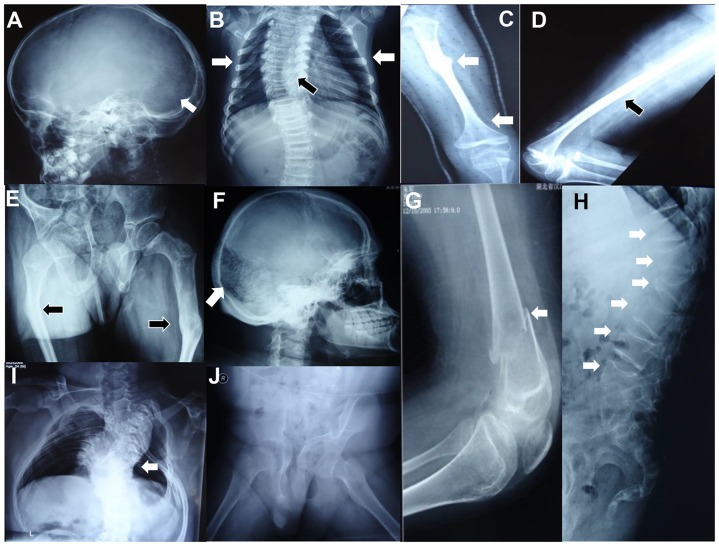
Radiological findings in the two probands with Bruck syndrome. For proband 1, (A) occipital wormian bone (white arrow); (B) scoliosis (black arrow) and thoracic cage collapse (white arrows); (C)–(D) slender femur with thin cortices (black arrow), metaphyseal enlargement of distal femur (white arrow) and old fracture (white arrow); (E) old fractures and bending of femora (black arrows), severe deformity of pelvis and osteoporosis. For proband 2, (F) occipital wormian bone (white arrow); (G) embedded fracture of right distal femur (white arrow); (H) multiple vertebral compression fractures (white arrows) and ithyokyphosis; (I) rotation kyphoscoliosis (white arrow), indistinct ribs and thoracic cage collapse; (J) severe deformity of pelvis and osteoporosis.

The proband was a 9-year-old boy, who was born through full-term normal delivery. The birth weight was 3500 g (the 50th centile) with unknown body length and head circumference. The boy had congenital symmetrical flexion deformity of knees. Bilateral thumb-in-palm deformities were presented at birth and then were corrected by an orthopedic surgery at the age of five years. When he was 1.5, 6.0, 8.5 and 8.8 years old, multiple bone fractures occurred at right femur for one time and at left femur for three times following trivial trauma. Although the fractures healed uneventfully under conservative treatment, he was still unable to stand and walk because of the severe contractures of knees and curvature deformities of femora. Intellect and other milestones of development were normal. The family history was non-contributory. Physical examination showed that he had short stature with deformities of knees. His height was 2.3 SD less than the average. He had no blue sclerae, hearing loss, dentinogenesis imperfecta or clubfoot. Bilateral knee arthrogryposis with pterygia and small joint laxity were shown in Figure 1A–C. The X-ray films revealed severe generalized osteoporosis with presence of wormian bone in cranium, flattening of vertebral bodies with scoliosis, thoracic cage collapse, deformity of pelvis, bowing femora with old healed fractures, slender long bone with thin cortices, metaphyseal enlargement of distal femur, narrowed joint space of knees (Figure 2A–E). We gave the patient alendronate (ALN) (Fosamax, Merck Sharp & Dohme Pharmaceutical Co., LTD) 70 mg weekly for 3 months. Because of adverse effects of gastrointestinal tract, he turned to accept infusion of zoledronic acid (ZOL) (Aclasta, Novartis Pharma Stein AG) at dose of 5 mg yearly. 500 mg calcium plus 200 IU vitamin D_3_ was supplemented daily.

### Family 2

The proband was a 22-year-old man. He was the only child of a non-consanguineous couple. He was born at full-term with footling presentation and suffered from a right femoral fracture during difficult delivery. Since then, nearly twenty times of fractures occurred at his bilateral ribs, right humerus and femora. Notably, the patient presented left pes equinovarus and mild limitation of joint movement of knees and right elbow. Camptodactyly was found at right thumb, fourth to fifth finger, left third to fourth fingers and fourth toes (Figure 1D–H). He had apparent kyphoscoliosis (Figure 1I). His stature was 3.6SD shorter than the average. He could not walk independently because of contractures of knees and discrepancy of lower limb length. He had normal sclerae, hearing, dentition and intelligence. His parents were healthy without family history of OI. The X-ray films also revealed severe generalized osteoporosis with wormian bone in cranium, multiple vertebral compression fractures with rotation kyphoscoliosis, collapsed right thoracic cage, deformity of pelvis, slender long bone with thin cortices (Figure 2F–J). He took 70 mg ALN weekly for 16 months. Since bone mineral density (BMD) gain was not obvious, he accepted infusion of 5 mg ZOL yearly. Supplementation of 500 mg calcium and 200 IU vitamin D_3_ was given daily.

100 unaffected, unrelated, ethnically matched, healthy subjects were recruited from the outpatients in the department of endocrinology, PUMCH. This study was approved by the Ethic Committee of PUMCH. Signed informed consents were obtained from all subjects included in this study or from their parents.

### Mutation Analysis

Genomic DNA of the probands, their parents and ethnically matched control was extracted from peripheral leukocytes with a QIAamp DNA Mini Kit (50) (Qiagen, Germany). All exons of *FKBP10* and *PLOD2*, exon–intron junctions were amplified by polymerase chain reaction (PCR) in 23 reactions. Primers were designed using the software Oligo 7.0 (Table S1). Taq DNA polymerase (Biomed, China) and its standard buffer were used in all reactions under the following conditions: initial denaturation at 95°C for 2 min, followed by 35 cycles at 95°C for 30 s, 53–63°C for 30 s, and 72°C for 45/90 s. Direct sequencing reactions of PCR products were performed using BigDye Terminators Cycle Sequencing Ready Reaction Kit, version 3.1 (Applied Biosystems), and analyzed with an ABI 3130 automatic sequencer (Applied Biosystems) using standard methods. The results of sequencing were compared with the reference nucleotide sequence of *FKBP10* (NM_021939.3) and *PLOD2* (NM_182943.2). Genetic mutations identified in our patients were submitted to the OI database (https://oi.gene.le.ac.uk).

### Assessment of Effect and Safety of Bisphosphonates

New fracture was determined by medical history and X-ray films of bone. BMD at lumbar spine 2–4 (L2–L4), femoral neck (FN) and total hip (TH) was measured at baseline and after 6 and 12 months of treatment by dual-energy x-ray absorptiometry (DXA, Lunar Prodigy Advance, GE Healthcare, USA) according to the manufacturer's protocol. The BMD Z scores were calculated according to BMD of the age matched boys in Beijing, China [12], [13]. BMD of the first proband's parents was also measured.

Serum levels of calcium, phosphate, alanine aminotransferase (ALT) and creatinine were detected by automatic analyzer. Serum levels of β-isomerized carboxy-telopeptide of type I collagen (β-CTX, bone resorption marker), total alkaline phosphatase (TALP, bone formation marker), 25 hydroxy vitamin D (25(OH)D, marker of vitamin D nutritional status) and intact parathyroid hormone (PTH) were measured by automated electrochemiluminescence system (E170; Roche Diagnostics, Switzerland). All biochemical tests were performed in the clinical central laboratory of PUMCH.

All adverse events were recorded in detail during the follow-up. Safety of the treatment was assessed by the records of adverse effects, physical examination and assessment of hematologic biochemical markers of liver and kidney functions.

## Results

### Mutations Identified in *FKBP10* and *PLOD2*

**Figure 3:**
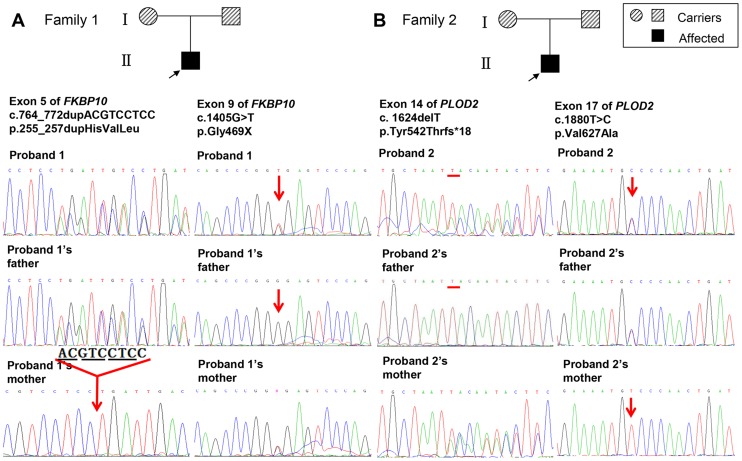
Pedigree of the two families displaying Bruck syndrome and mutation analysis. Probands are indicated by the arrows. Black symbols indicate individuals with Bruck syndrome; shadow symbols represent carriers. (A) In proband 1, novel compound heterozygous mutations of *FKBP10* were identified as: c.764_772dupACGTCCTCC (p.255_257dupHisValLeu) in exon 5 and c.1405G>T (p.Gly469X) in exon 9. (B) In proband 2, novel compound heterozygous mutations of *PLOD2* were identified as: c. 1624delT (p.Tyr542Thrfs*18) in exon 14 and c.1880T>C (p.Val627Ala) in exon 17. The probands' parents were both asymptomatic heterozygous carriers for the compound mutations, respectively.

**Figure 4:**
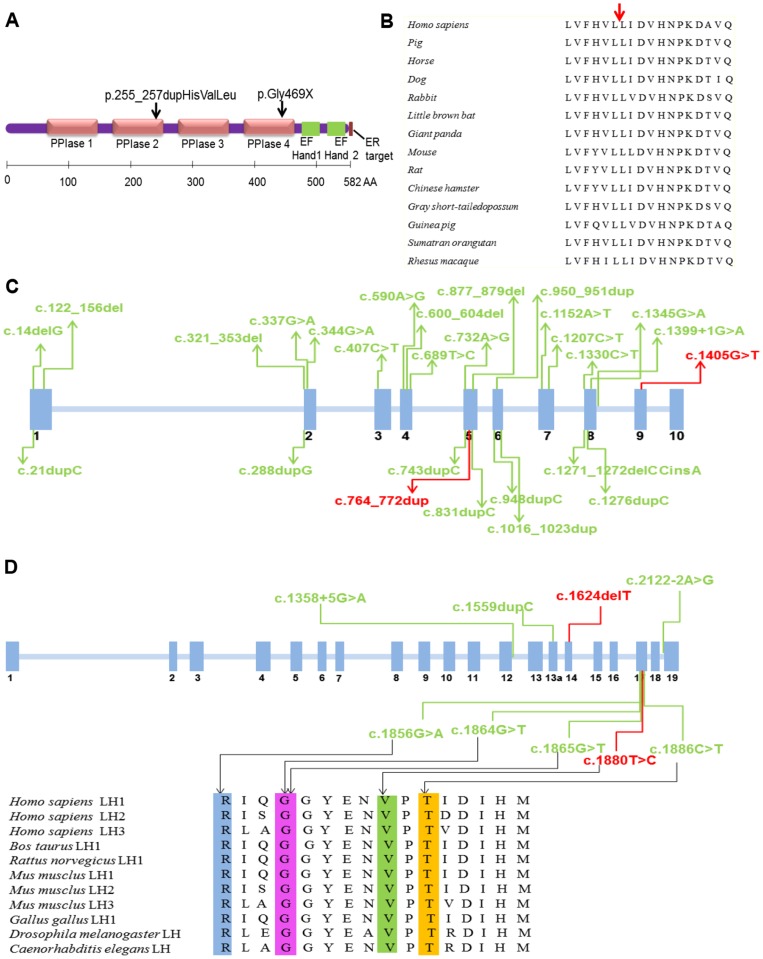
Mutation information of *FKBP10* and *PLOD2*. (A) Representation of FKBP65 with the location of mutations in proband 1 (black arrows). (B) The whole stretch sequence around Leu258 is highly conserved in FKBP65 among 14 different species. Red arrow indicates position of p.255_257dupHisValLeu mutation. (C) Blue boxes indicate all exons of *FKBP10*. Mutations were identified in *FKBP10* in patients with Bruck syndrome and osteogenesis imperfecta. The two kinds of mutations in our study are shown in red. (D) Blue boxes indicate all exons of *PLOD2*. Mutations were identified in *PLOD2* in patients with Bruck syndrome and osteogenesis imperfecta. The two kinds of mutations in this study are shown in red. Mutation in exon 17 of proband 2 will lead to change of amino acid in lysyl hydroxylase at highly conserved among 11 different species.

For the first proband, DNA sequence analysis indicated compound heterozygous mutations in *FKBP10* gene: 9 base pairs duplication (c.764_772dup ACGTCCTCC) in exon 5 resulting in three amino acids duplication (p.255_257dupHisValLeu) and c.1405G>T transition in exon 9 leading to the substitution of glycine at position 469 by premature termination codon (p.Gly469X) (Figure 3A). This duplication occurred in the second peptidyl-prolyl cis-trans isomerase (PPIase) domain of FKBP65 (Figure 4A), among a region of highly conserved residues (Figure 4B). The nonsense mutation occurred in the fourth PPIase domain of FKBP65 (Figure 4A), generating a premature truncation of the protein. The parents were heterozygous carriers for one of the compound mutations, respectively.

For the second proband, compound heterozygous mutations in *PLOD2* gene were identified in DNA sequence analysis: one base pairs deletion (c.1624delT) in exon 14 resulting in frame shift (p.Tyr542Thrfs*18) and c.1880T>C transition in exon 17 leading to the substitution of valine at position 627 by alanine (p.Val627Ala) (Figure 3B). His parents were heterozygous carriers for one of the compound mutations, respectively.

The four kinds of novel mutations were absent in the 100 unrelated control subjects and were not classed as polymorphisms in all public databases.

### Effect and Safety of Bisphosphonates

At baseline, the first proband had increased level of β-CTX and normal levels of calcium, phosphate, PTH and TALP (Table 1). BMD and Z scores at L2–L4, FN and TH were extremely low (Table 1). After 9 months treatment of BPs, serum β-CTX level was decreased from 1.3 ng/mL to 1.0 ng/mL and serum TALP level was decreased from 353 U/L to 194 U/L (Table 1). BMD at L2–L4, FN and TH was significantly increased by 125.5%, 31.4%, 49.3% compared to baseline, respectively (Figure S1A). The Z scores of BMD at all above sites were increased from −4.1, −3.9 and −2.9 of baseline to −2.5, −3.1 and −1.2, respectively (Figure S1B). However, two times of new fractures at different positions of left femur recurred after 2 weeks and 10 months of ZOL treatment. The laboratory results and BMD of his patients were all normal. His father's BMD Z scores at L2–L4, FN and TH were −0.6, 0.0 and −0.7, respectively. His mother's BMD Z scores at above sites were −1.1, 0.0 and −0.1, respectively.

**Table 1:** Clinical characteristics of the two patients with BS.

	Proband 1		Proband 2		
Characteristics	Baseline	9M visit	Baseline	23M visit	Reference values
Age (years)	9	10	22	24	
Height (cm)	120	120	150	150	
Weight (kg)	19.5	20.0	79	79	
Serum calcium (mmol/L)	2.39	2.34	2.40	2.36	2.13–2.70
Serum phosphate (mmol/L)	1.68	1.86	1.05	0.95	0.81–1.45
Serum TALP (U/L)	353	194	124	113	30–120 (adult)
					42–390 (child)
Serum β-CTX (ng/mL)	1.3	1.0	0.4	0.3	0.26–0.512
Serum creatinine (µmol/L)	24	25	46	55	59–104 (adult)
					18–62 (child)
Serum ALT (U/L)	10	10	77	64	5–40
Serum intact PTH (pg/mL)	18.7		37.5		12–65
Serum 25(OH)D (ng/mL)	25.4		12.7		
L2–L4 BMD (mg/cm^2^)(Z score)	216 (−4.1)	487 (−2.5)	1186 (0.2)	1324 (1.7)	
FN BMD (mg/cm^2^) (Z score)	338 (−3.9)	444 (−3.1)	877 (−1.2)	1063 (0.7)	
TH BMD (mg/cm^2^) (Z score)	469 (−2.9)	700 (−1.2)	906 (−0.8)	941 (−0.4)	

BS, Bruck syndrome; TALP, total alkaline phosphatase; β-CTX, β-isomerized carboxy-telopeptide of type I collagen; ALT, alanine aminotransferase; PTH, parathyroid hormone; 25(OH)D, 25 hydroxy vitamin D; L2–L4, lumbar spine 2–4; FN, femoral neck; TH, total hip.

At baseline, the second proband had mildly increased TALP and ALT levels, normal β-CTX level (Table 2). The DXA films of spine showed the severe kyphoscoliosis with dim shapes of ribs (Figure S2C). BMD at proximal femur was low. BMD at lumber spine was normal. However, severe deformity of lumbar spine would lead to bias of measurement. After 23 months treatment of BPs, serum β-CTX level was decreased from 0.4 ng/mL to 0.3 ng/mL and serum TALP level was decreased from 124 U/L to 113 U/L. BMD at L2–L4, FN and TH was significantly increased by 11.6%, 21.2%, 3.9% compared to baseline, respectively (Figure S2A). The Z scores of BMD at all above sites were increased from 0.2, −1.2 and −0.8 of baseline to 1.7, 0.7 and −0.4, respectively (Figure S2B). No new fracture occurred during period of BPs treatment.

**Table 2:** Clinical, radiological and genetic information of patients with BS.

Patients	Proband 1 in Chinese	Proband 2 in Chinese	Patients with *FKBP10* mutations [4], [7], [9], [15], [16], [23], [26]–[29]	Patients with *PLOD2* mutations [5], [7], [25]
Causitive mutations	*FKBP10* c.764_772dupACGTCCTCC, c.1405G>T	*PLOD2* c.1624delT, c.1880T>C	Mostly duplication mutations	Mostly substitutionmutations
Race	Han	Han	Variable	Variable
Sex	Male	Male	Both	Both
Positive family history	-	-	+/-	+/−
Consanguineous pedigree	-	-	Most	Most
Postnatal short stature	+	+	+	+
First fracture age	18 months	At birth	Early infancy	Early infancy
Times of fracture	6	nearly 20	Variable	Variable
Congenital joint contractures	Knees	Knees, right elbow	Mostly knees, elbows and ankles; wrists and hips also reported	Mostly knees and elbows; wrists and ankles also reported
Camptodactyly	thumbs	4th toes, right thumb and 4–5 fingers, left 3–4fingers	8/38 cases	2/6 cases
Clubfoot	-	left	15/38 cases	5/6 cases
Scoliosis/kyphoscoliosis	+	+	Most	Most
Blue sclerae	-	-	- (almost all)	- (almost all)
Dentinogenesis imperfecta	-	-	-	-
Wormian bones	+	+	Most	Most
Mobility	Difficulty in walking with limping			

BS, Bruck syndrome; N.A, not available.

The ability of daily activities of the two probands was significantly improved after BPs treatment. Both of the patients had mild fever and muscle soreness in the second day after infusion of ZOL, with the highest body temperature of 37.3 and 37.8 centigrade respectively. The adverse effects were in remission after 2 to 3 days spontaneously without special management. No osteonecrosis of jaw or other adverse effects were observed.

## Discussion

BS is an exceedingly rare form of OI, which presents the concurrence of bone fragility and congenital joint contractures in an individual. In 1897, Bruck reported a man with bone fragility and joint contractures, which was the first description of this syndrome [14]. We reported two new patients with this rare disease for the first time in Chinese. They presented with recurrent fractures, multiple congenital arthrogryposis, postnatal short stature and progressive scoliosis, but without blue sclerae, dentinogenesis imperfecta or hearing loss. The causative mutations were identified as novel compound heterozygous mutations in *FKBP10* and *PLOD2*, respectively, and their parents are asymptomatic heterozygous carriers for one of the compound mutations.

*FKBP10* and *PLOD2* locate at 17q2 and 3q24, respectively. They are reported to be causative loci for moderate to severe isolated recessive OI, as well as BS [7], [15], [16]. 25 kinds of mutations have been identified over whole exons of *FKBP10* gene, which mainly focus on exon 5 (32.26%) and exon 6 (24.73%) (Figure 4C) (https://oi.gene.le.ac.uk). Duplication mutations (54.84%) in *FKBP10* resulting in frame shift of the amino acid sequence are the majority cause of recessive OI with or without joint contractures (https://oi.gene.le.ac.uk). Only 7 kinds of mutations in *PLOD2* have been identified which mainly focus on exon 17 (60%) (Figure 4D)(https://oi.gene.le.ac.uk). Substitution mutations (80%) in *PLOD2* are the majority causes of recessive OI with or without joint contractures. Deletion mutation in exon 14 of *PLOD2* in our patients is identified for the first time (https://oi.gene.le.ac.uk). In our patients, compound heterozygous mutations of c.764_772dupACGTCCTCC in exon 5 and c.1405G>T in exon 9 of *FKBP10*, and c.1624delT in exon 14 and c.1880T>C in exon 17 of *PLOD2* are all novel mutations of causative gene of BS.

FKBP65 has four PPIase domains that catalyze the interconversion of cis/trans isomers of peptidyl-prolyl bonds in proteins containing proline [17]. Since approximately one-sixth of the collagen sequence consists of proline residues, this conversion is the rate-limiting step in the folding process of nascent proteins [18]. FKBP65 prevents premature cross-links between procollagen chains and assists proper registration, folding into the collagen triple helix, and subsequent trafficking [19], [20]. Moreover, FKBP65 could modulate type I collagen cross-linking indirectly and is crucial for the stability or activity of LH2 [15], [19], [21]. Inactivating mutations of *FKBP10* could lead to loss function of FKBP65, marked diminution of lysyl hydroxylation, instable secreted type I procollagen similar to mutations in *SERPINH1* [22]–[24]. The compound variants of *FKBP10* in our patient affect the second and fourth PPIase domain, which will reduce folding and cross-linking of type I procollagen.

Fibrillar collagen molecules in tissues are connected through lysyl- and hydroxylysyl-derived cross-links. The involved lysyl residues locate in the amino-terminal and carboxyl-terminal telopeptides and two sites in the triple-helical domain. LH2 is highly expressed in bone and is mainly responsible for hydroxylation of the triple-helical cross-linking lysyl residues. In this study, mutation c. 1624delT in exon 14 of *PLOD2* results in frame shift. Mutation c.1880T>C of *PLOD2* causes the substitution of valine by alanine at position 627, which is close to previously identified variants of p.Arg619His(c.1856G>A), p.Gly622Cys(c.1864G>T), p.Gly622Val(c.1865G>T) and p.Thr629Ile(c.1886C>T) in patients with BS (Figure 4D) [5], [7], [25]. This region is proposed to be an important functional domain of LH2 enzyme and is highly conserved throughout the species [25]. Moreover, the missense mutation may lead to damage of protein predicted by SIFT online software (http://sift.jcvi.org/). Impaired function of LH2 will lead to abnormal hydroxylation of the triple-helical cross-linking lysyl residues, and then will affect the installment and secretion of the collagen fibers from osteoblasts.

The phenotypes of Chinese patients are generally similar to clinical spectrum in other ethnical patients with BS (Table 2). Mutations of either *FKBP10* or *PLOD2* gene lead to congenital joint contractures of knees, elbows and other joints. Talipes and camptodactyly seem to be the more common sign of patients with *PLOD2* mutations. However, since there are few patients recorded with BS, the genotype-phenotype correlation still needs to be investigated in more patients. The overlapped phenotype induced by *FKBP10* and *PLOD2* mutations possibly attribute to the common mechanisms affecting collagen cross-link formation through diminution of telopeptide lysyl hydroxylation. However, age of initial fracture, times of fracture and limitation of joints movement are variable among patients with BS. In order to elucidate the detail mechanism of BS caused by *FKBP10* and *PLOD2* mutations, we should further analyze the molecular stability and post-translational modification of type I procollagen through culturing fibroblasts or osteoblasts of the patients with BS using Western blot analysis or immunocytochemical analysis of proteins. The C18 reverse-phase HPLC is helpful to complete quantitative analysis of the cross-link of type I collagen [15].

Effective treatment for BS is still deficient. BPs are widely used to treat osteoporosis through potent inhibition of osteoclasts [30]. Since increasing osteoclast activity contributes to pathogenesis of OI [31], pamidronate, risedronate, ALN or ZOL are demonstrated to reduce fracture incidence and improve musculoskeletal function of patients with OI [32]–[36]. We chose ZOL to treat this rare disease because of its convenience and no gastrointestinal adverse effect. Significant increases in areal BMD and its Z scores at lumbar spine and proximal hip in patients with BS were observed after treatment of ZOL. The bone resorption biomarker level was decreased and the ability of daily activities of the patients was improved after treatment of BPs. ZOL was generally well tolerated to BPs. However, the efficacy and safety of BPs on BS still needs to be further confirmed in large sampled clinical study.

## Conclusions

BS is an extremely rare, autosomal recessively inherited, moderate or severe type of OI, which is characterized by congenital joint contracture, multiple fractures and postnatal short stature. Novel compound heterozygous mutations c.764_772dupACGTCCTCC (p.255_257dupHisValLeu) and c.1405G>T (p.Gly469X) in *FKBP10*, c.1624delT (p.Tyr542Thrfs*18) and c.1880T>C (p.Val627Ala) in *PLOD2* are the new genetic mechanisms of BS. Moreover, intravenous zoledronic acid is probably an effective treatment option for patients with BS.

## Supporting Information

Figure S1 **Effects of bisphosphonates on proband 1 with Bruck syndrome.** (A)–(B) BMD and Z score at all sites were significantly increased after 9 months of bisphosphonates treatment. (C) Bone morphology was improved in spine and right femur after 9 months of treatment, which was showed in images of DXA. BMD, bone mineral density; DXA, dual-energy x-ray absorptiometry; L2–L4, lumbar spine 2–4; FN, femoral neck; TH, total hip; ALN, alendronate; ZOL, zoledronate acid.
(TIF)

Figure S2 **Effects of bisphosphonates on proband 2 with Bruck syndrome.** (A)–(B) BMD and Z score were increased at L2–L4 after bisphosphonates treatment. BMD and Z score at proximal femur was decreased after 16 months of ALN treatment but was significantly increased after 7 months of ZOL treatment. (C) In images of DXA, color of lumbar spine became darker after 23 months of treatment. No significant change in image of proximal femur was observed after treatment. BMD, bone mineral density; DXA, dual-energy x-ray absorptiometry; L2–L4, lumbar spine 2–4; FN, femoral neck; TH, total hip; ALN, alendronate; ZOL, zoledronate acid.
(TIF)

Table S1 **List of primers for PCR-amplification of all exons and exon–intron junctions of** ***FKBP10*** **and** ***PLOD2*** **.**
(DOCX)
