# Supplementary figures and images for: Novel Mutations in FKBP10 and PLOD2 Cause Rare Bruck Syndrome in Chinese Patients
Source: PLoS One. 2014 Sep 19;9(9):e107594. doi: 10.1371/journal.pone.0107594 (PMC4169569; doi:10.1371/journal.pone.0107594)

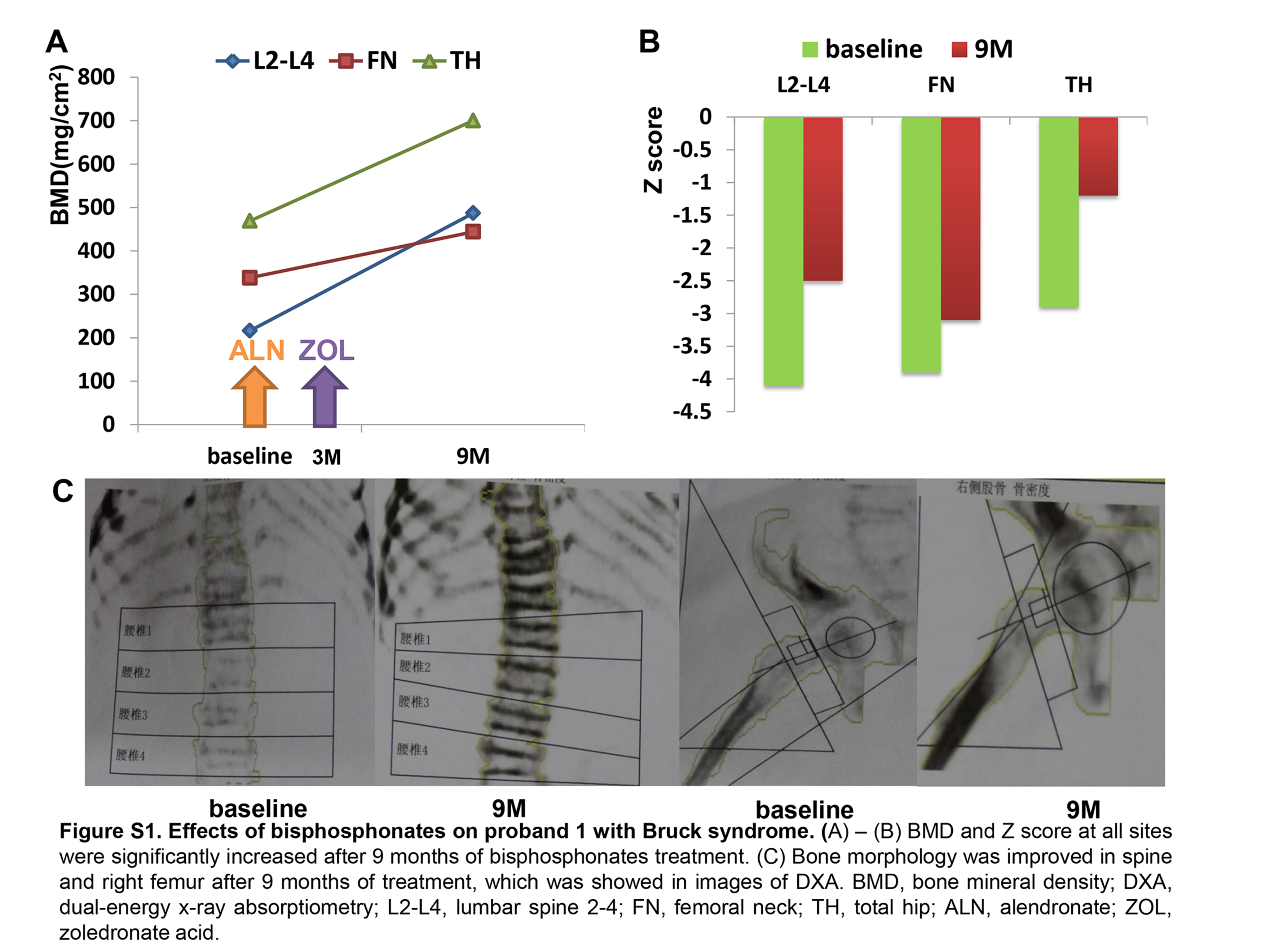

Supplement: Figure S1 — Effects of bisphosphonates on proband 1 with Bruck syndrome. (A)–(B) BMD and Z score at all sites were significantly increased after 9 months of bisphosphonates treatment. (C) Bone morphology was improved in spine and right femur after 9 months of treatment, which was showed in images of DXA. BMD, bone mineral density; DXA, dual-energy x-ray absorptiometry; L2–L4, lumbar spine 2–4; FN, femoral neck; TH, total hip; ALN, alendronate; ZOL, zoledronate acid. (TIF) [file pone.0107594.s001.tif]

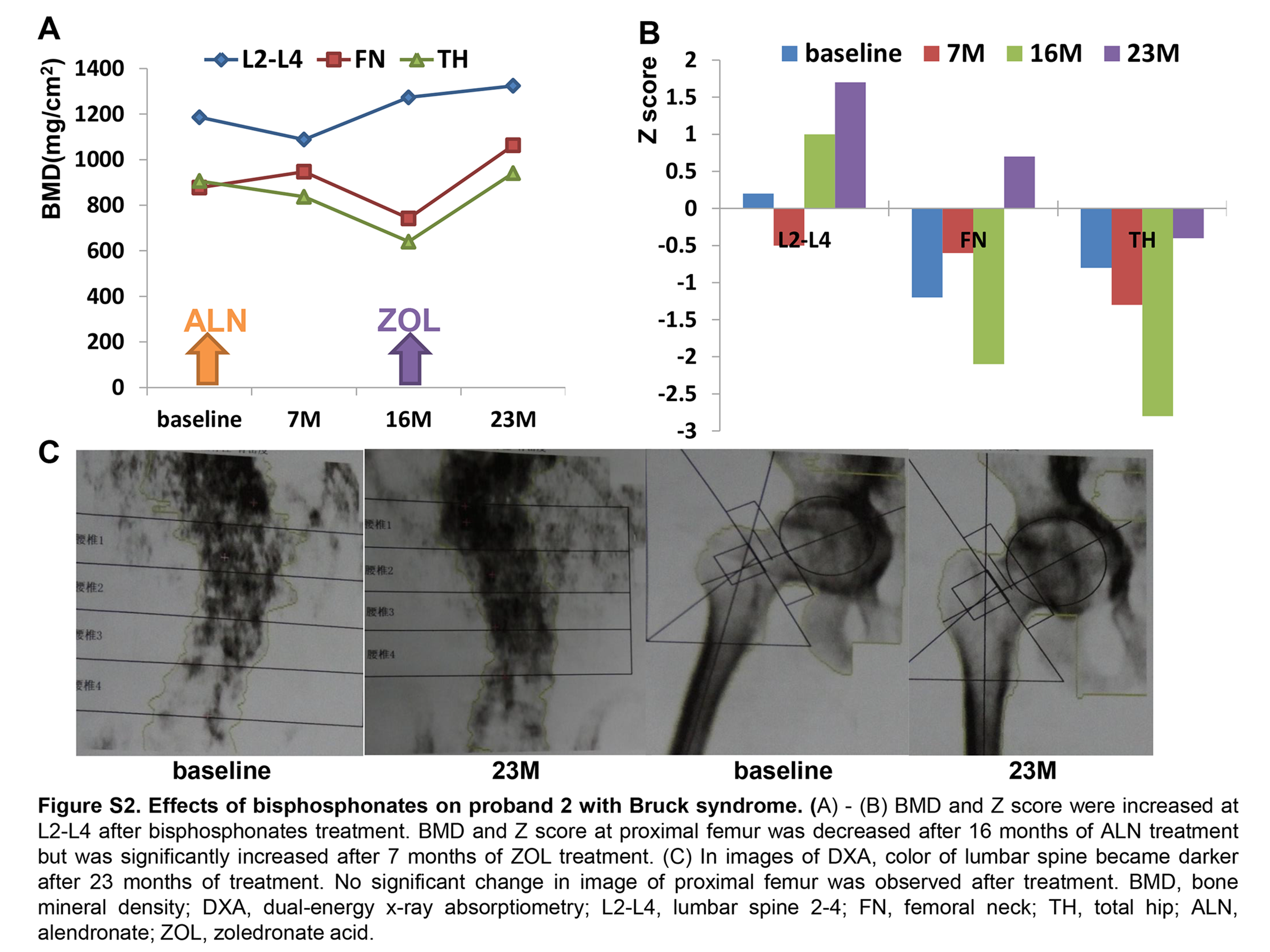

Supplement: Figure S2 — Effects of bisphosphonates on proband 2 with Bruck syndrome. (A)–(B) BMD and Z score were increased at L2–L4 after bisphosphonates treatment. BMD and Z score at proximal femur was decreased after 16 months of ALN treatment but was significantly increased after 7 months of ZOL treatment. (C) In images of DXA, color of lumbar spine became darker after 23 months of treatment. No significant change in image of proximal femur was observed after treatment. BMD, bone mineral density; DXA, dual-energy x-ray absorptiometry; L2–L4, lumbar spine 2–4; FN, femoral neck; TH, total hip; ALN, alendronate; ZOL, zoledronate acid. (TIF) [file pone.0107594.s002.tif]
