# Supplementary material for: Novel Mutations in FKBP10 and PLOD2 Cause Rare Bruck Syndrome in Chinese Patients
Source: PLoS One. 2014 Sep 19;9(9):e107594. doi: 10.1371/journal.pone.0107594 (PMC4169569; doi:10.1371/journal.pone.0107594)
Supplement: Table S1 — List of primers for PCR-amplification of all exons and exon–intron junctions of FKBP10 and PLOD2 . (DOCX) [file pone.0107594.s003.docx]

**Table S1** List of primers for PCR-amplification of all exons and exon–intron junctions of *FKBP10* and *PLOD2*.

| Exon | | Forward (5’→3’) | | Reverse (5’→3’) | Annealing temperature |
| --- | --- | --- | --- | --- | --- |
| ***FKBP10*** | | |  | |  |
| 1 | GCCTCCCTCAGACTCTAACACA | | ATACAGCCCTGGTAACTACACA | | 61°C 45’’ |
| 2-3 | TGCCTTTCACAACAGTCCGTTC | | TGCCCTTGATCAGCCAACCAG | | 61°C 90’’ |
| 4-5 | TGCCACCCTCTACTTCGATG | | GATGATGTAACCCTGCCCGAT | | 61°C 90’’ |
| 6-7 | CGGCACCCTCTTCGATTCCA | | CTCATGGCTCCCTGCGTCA | | 61°C 90’’ |
| 8-9 | CTATTTCACAGAGGGGAAACTGAC | | CCCCGCACCCTGTAACTCA | | 61°C 90’’ |
| 10 | AGGCTGTTCCCTACCTGA | | CAGGCCCCAAATTGGCTTT | | 61°C 45’’ |
| ***PLOD2*** | | |  | |  |
| 1 | CACCGACGACCTCACTCAG | | GGAGTACTGCCTCGTCCATC | | 63°C 45’’ |
| 2 | TTTTGCGTTGAAATAATTGTCA | | CAGAATACTTTGGCTATCCCC | | 53°C 45’’ |
| 3 | AAATACTTTCTGGGTCTACTTC | | CCTTGTGATCCAGCTGCCTAG | | 53°C 45’’ |
| 4 | GCTGTTTCAGAATTTCAATTTGG | | AACTTATTATCCTAGAGCAA | | 53°C 45’’ |
| 5 | GTAAATCTGTTTCCCTTAGGAGC | | TGCCTGATCCAACCAAGTTCG | | 53°C 45’’ |
| 6 | CTTTAAGTGTATTTTGCAATGAG | | TCTAATACTCTGGGTAATTCGT | | 53°C 45’’ |
| 7 | CCCTAAAAGACAGACGTGGA | | TGTTAACTTAGAAAACGGCAAG | | 53°C 45’’ |
| 8 | TTTTGATCATCCTTTCCGGTA | | CCAGCACAGATAAAATTAGTCAA | | 53°C 45’’ |
| 9 | AGTATTTGGTTTCTTAGACGAA | | TTTAAGGCCCTTTGAATCTCC | | 53°C 45’’ |
| 10 | TCTAAGATTTCTAGGCTACAGG | | ACACAGTCTAAGTTGGCTA | | 53°C 45’’ |
| 11 | GAATATCCGTGTTAAATTGACC | | GTATCATAATTTCACCTAGCTT | | 53°C 45’’ |
| 12 | TTCATTTAATATAGGTTCCGAA | | ATATATTATCTCTTAGTATTCCCC | | 53°C 45’’ |
| 13-13a | AAATCTCATTAAATATAGTGT | | ATCAACATTTTACCAAGC | | 53°C 90’’ |
| 14 | AAAATATTTCTGCTTTCAAACAGG | | TTTCCAAATTCATTCCGACCA | | 53°C 45’’ |
| 15-16 | ATAACCCCATCGTGAGTCAAGG | | TTCAGAGCCAGATAACATTTGCC | | 53°C 90’’ |
| 17 | ACCTTACATTACTTTGTTTCGTC | | AAAGTCTTCTCCCACGTT | | 53°C 45’’ |
| 18-19 | GTCTTTGCAGGCTATTATACGAA | | TCTCAGAGGCAACAAAGCATA | | 53°C 45’’ |
